# Gecko Adhesion on Wet and Dry Patterned Substrates

**DOI:** 10.1371/journal.pone.0145756

**Published:** 2015-12-22

**Authors:** Alyssa Y. Stark, Amanda M. Palecek, Clayton W. Argenbright, Craig Bernard, Anthony B. Brennan, Peter H. Niewiarowski, Ali Dhinojwala

**Affiliations:** 1 Integrated Bioscience Program, The University of Akron, Akron, Ohio, United States of America; 2 Department of Materials Science and Engineering, University of Florida, Gainesville, Florida, United States of America; 3 Department of Polymer Science, The University of Akron, Akron, Ohio, United States of America; Harbin Institute of Technology, CHINA

## Abstract

Perhaps one of the most astounding characteristics of the gecko adhesive system is its versatility. Geckos can locomote across complex substrates in a variety of conditions with apparent ease. In contrast, many of our synthetic pressure sensitive adhesives fail on substrates that are dirty, wet or rough. Although many studies have investigated the effect of environmental challenges on performance, the interaction of multiple, potentially compromising variables is studied less often. Here we focus on substrate structure and surface water, both of which are highly relevant to the biological system and to synthetic design. To do this we utilized a highly controlled, patterned substrate (Sharklet^®^, by Sharklet^®^ Technologies Inc.). This allowed us to test independently and jointly the effects of reduced surface area substrates, with a defined pattern, on adhesion in both air and water. Our results show that adhesion is not significantly impaired in air, whereas surface area and pattern significantly affect adhesion in water. These findings highlight the need to study multiple parameters that are relevant to the gecko adhesive system to further improve our understanding of the biological system and to design better, more versatile synthetics.

## Introduction

Geckos have fascinated scientists and the lay person for centuries due to their ability to stick repeatedly to substrates that are otherwise considered challenging or even impossible for current synthetics [1]. Challenges such as dirt and dust, surface chemistry and structure, and even water, can often be circumvented by this versatile natural adhesive system [2–9]. Although studies focusing on testing gecko adhesion on such substrates are increasing, there still remains an important gap. This gap is highlighted when we consider geckos in their natural environment. In contrast to many current studies, geckos are unlikely to encounter only one or two "challenges" at a time, and rather, are likely required to retain function on substrates that present multiple challenges. For instance, consider a gecko moving across the surface of a tree branch. In one step this substrate is both dirty and rough at multiple scales. Therefore a gecko's foot must conform to each of the roughness scales (nanometer, micrometer, millimeter and even centimeter-level), while at the same time self-cleaning dirt particles to facilitate reliable adhesive performance. Furthermore, consider the environment in which adhesion takes place. In the tropics, factors such as temperature, humidity and surface water can be highly variable and often extreme. All of these parameters have been shown to significantly affect whole animal gecko adhesion [5, 10–13], and in the case of temperature and humidity this is a joint effect [10]. Currently most experimental designs decouple specific parameters and independently test their affect on adhesion. Far less work has been done considering multiple joint affects, specifically with the whole animal [5, 9, 10, 14]. In this study we consider joint effects of substrate condition on whole animal adhesion. We focus on three specific parameters, which are likely to interact, and are relevant to the gecko's natural environment.

The first parameter relates to the hierarchical nature of the gecko adhesive system. The gecko adhesive system consists of soft, flattened toe pads (in many species) that are supported by compliant muscle, tissue and venous structure [15–18]. In turn, each toe consists of rows of elevated skin folds called lamellae, which provide further compliance at the millimeter-level scale [16]. Each lamellae is covered by small hair-like structures called setae, which branch and terminate into flattened tips called spatulae [16, 19–21]. The setae and the spatulae scale on the micrometer and nanometer scales respectively [22], and their combined hierarchy further reduces overall modulus of the system [23]. Thus, when pressing onto a rough or structured substrate like bark, the gecko's foot conforms at multiple scales to make close contact with the substrate. It is this close contact that allows weak intermolecular van der Waals forces to dominate and the gecko to stick and peel easily from a substrate without the need for gluey secretions [2]. While the hierarchical design is certainly versatile, there are limitations. Specifically, it appears that substrate asperities at scales similar to components of the adhesive system significantly reduce adhesion [6, 24], although this may not always be the case [25]. It is likely limitation occurs at all levels of hierarchy (i.e. at the setal level and the toe pad level), in addition to spatular and lamellar-levels that have been investigated previously [6, 24]. At these limiting scales, sufficient contact area cannot be made to maintain adhesion because the surface asperities are neither small enough nor large enough for the specific level of hierarchy to conform to them [24]. Previous studies have focused on gecko adhesive performance on rough, uneven substrates [6–8, 24–27]. In most cases however, adhesion or locomotor performance was tested on heterogeneous substrates that had roughness scales at several levels, or did not have uniform distribution of roughness. The result is that multiple levels of hierarchy are tested, rather than one, and total surface area available for adhesion is not easily calculated (although see [28]).

The second parameter we were interested in testing poses another, similar challenge to gecko adhesion as substrate roughness. At high levels of humidity where water wets substrates or when substrates become wet from rain, adhesion should be negatively impacted. van der Waals adhesion relies on close contact with the substrate, and similar to the loss of surface area due to roughness asperities, surface water can limit the amount of contact (if any) a gecko makes with the substrate. On hydrophilic substrates this results in complete loss of adhesion, where sufficient adhesion is defined as the ability to support one's body weight [5, 11]. On hydrophobic substrates and intermediately wetting substrates however, geckos with dry toe pads can adhere just as strongly in water as they do in air [5]. It is hypothesized that the superhydrophobic toe pads achieve this by first, shielding the adhesive toe pad from water (i.e. wetting) and second, by pushing water out of the contact interface [5, 14]. Thus the interaction of the toe pad and the substrate is critical to adhesion on wet substrates. Considering the variability of substrates available to geckos in their natural environment, the interaction between water and the substrate can have important implications for ecology, behavior and even evolution of the gecko adhesive system.

Although the effect on adhesion, of both substrate roughness and surface water, has been investigated previously, we do not know how these two parameters affect gecko adhesion jointly. It is clear that some species of tree frogs are adapted to maintain adhesion on both wet and rough substrates [29]. This is also true for aquatic insects and fish that cling to rough submerged rocks [30–33]. Geckos however must adhere to rough substrates, like bark and leaves, that have thin layers of water or pools of water left behind from high humidity and rain. Furthermore, gecko adhesion relies on dry contact, instead of capillary adhesion, suction and gluey secretions like those of insects, fish and frogs, which complicates predictions about gecko adhesion to rough, wet substrates.

In an effort to investigate how geckos perform in multiple challenging conditions, we tested geckos on substrates that were both wet and had reduced surface area available for contact. Similar to examples on rough substrates, reduced surface area substrates should reduce adhesion (total surface area is not considered here but rather the surface area available for contact by the setae and spatula). Alternatively, substrates that are at least intermediately wetting (contact angle > ~80°) should not reduce adhesion when tested in water [5]. The interaction between a substrate submerged in water that also has reduced surface area is unknown. For instance, consider a substrate that has reduced surface area via channels or depressions. In water this substrate can either form air pockets in the asperities or form water-filled gaps where a gecko's foot can either contact a combination of the substrate and air or the substrate and water. The affect of this heterogeneous contact on adhesion is not clear. In addition, as mentioned previously, substrate asperities are often hard to control so the location of foot placement on heterogeneous substrates can increase variation of adhesive performance. To circumvent this, we took advantage of a unique substrate with controlled surface area and pattern.

**Fig 1:**
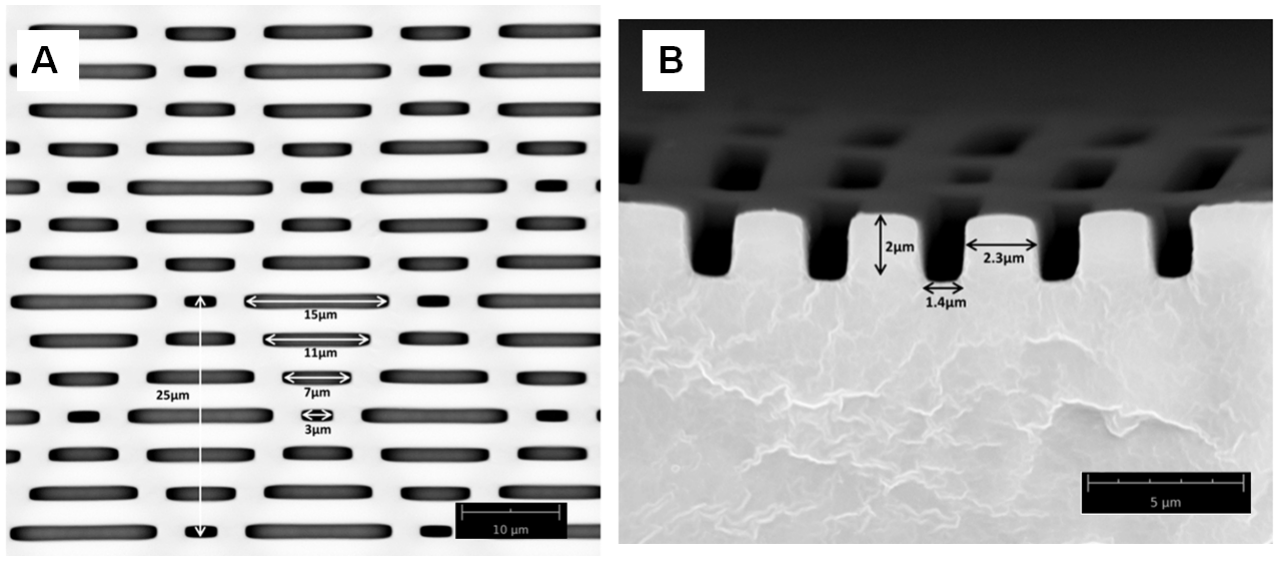
Dimensions of the Sharklet^®^ substrate. The patterned channel lengths (A) and depth (B) are shown.

**Fig 2:**
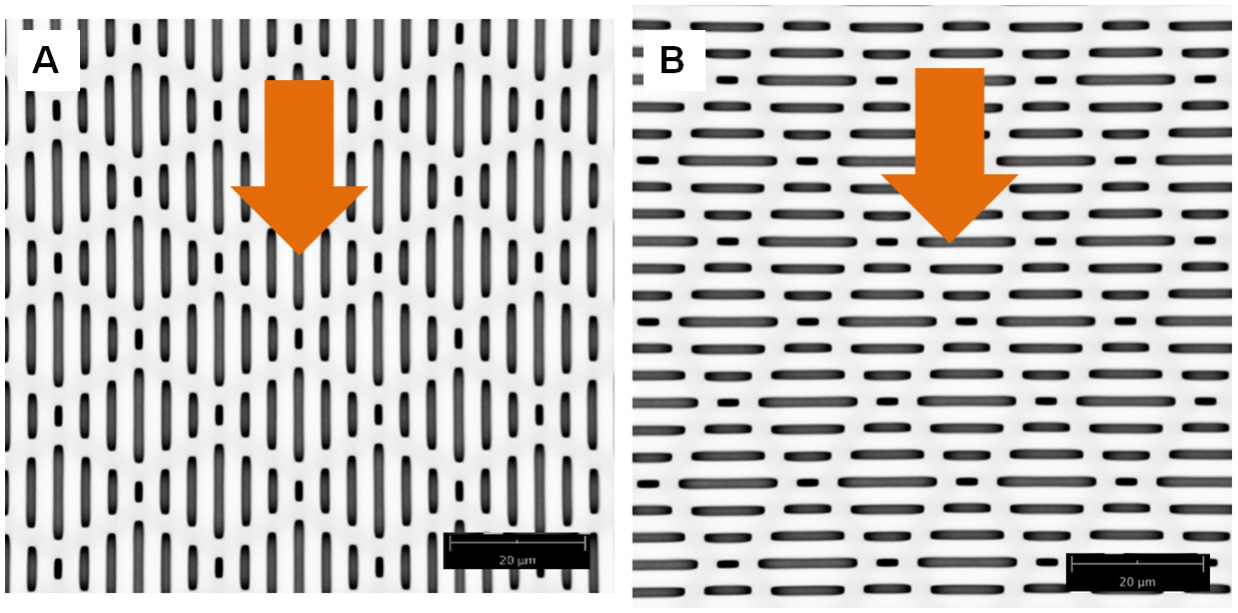
Image of Sharklet^®^ substrate orientation during shear adhesion tests. Geckos were either slid across the substrate parallel to the channels (A) or perpendicular (B). Arrows show direction of shear.

We used a patterned hydrophobic substrate with impressive anti-adhesion properties made by Sharklet^®^ Technologies Inc. Sharklet^®^ is a textured material that is specifically designed to inhibit bacterial and algal adhesion by way of structure alone [34, 35]. The structured material mimics natural anti-bacterial and anti-algal surfaces like shark skin [36]. Importantly for this study, the structure of this material is highly controlled and allows us to measure total available surface area for adhesion, which would not be possible for substrates with heterogeneous roughness (i.e. bark, sandpaper, cloth). The size scale of the surface structure is on the micrometer level, thus targets the adhesive setae. Furthermore, an additional, third parameter is available to us when testing gecko adhesion on Sharklet^®^ surfaces. This parameter is pattern. Sharklet^®^ has a distinct pattern that can be rotated to test shear adhesion of gecko feet on reduced surface area substrates that are either oriented in the direction of shear (parallel) or opposed (perpendicular). Figs 1 and 2 show the Sharklet^®^ substrate's patterned morphology and the direction of shear sliding the gecko makes during experiments (Figs 1 and 2).

The utilization of the Sharklet^®^ substrate allows us to control available surface area, test for the effect of pattern and test for the affect of environment (air or water). Thus we are able to investigate several unique hypotheses. First, we hypothesized that a smooth, non-patterned substrate will behave similarly to other smooth substrates tested previously. Specifically, on a smooth hydrophobic control substrate we hypothesized that geckos will adhere equally well in air and in water. Second, we hypothesized that on structured substrates, adhesion will be reduced in both air and in water and that pattern will have no affect on adhesion, as the robustness of the hierarchical adhesive system will be able to conform to either pattern equally well. While it is unlikely geckos routinely encounter uniform substrate structure and pattern in their natural environment, by controlling for these we will begin to assess both independently and jointly how such challenges as reduced available surface area, pattern and water affect gecko adhesion at the whole-animal scale.

## Materials and Methods

### Substrate Preparation and Characteristics

Xiameter T-2 Polydimethyl siloxane elastomer (PDMSe) was mixed in the ratio 10 parts base to 1 part curing agent, degassed under vacuum, and poured onto a patterned silicon wafer mold supplied by Sharklet^®^ Technologies, Inc. After curing in the mold for 24 hours the PDMSe was removed and trimmed resulting in a 4”x4” patterned PDMSe film. Six patterned films were assembled together and mounted onto an allyltrimethoxysilane functionalized glass plate using another thin layer of uncured PDMSe. The final result was an 8”x12” patterned PDMSe area attached to a glass plate. The smooth control substrate was cast as a thin layer of PDMSe between glass plates to yield a similar thickness as that of the patterned films (smooth = 1.06 ± 0.03mm, parallel = 0.99 ± 0.01mm and perpendicular = 1.08 ± 0.01mm).

Water contact angles were measured for each substrate at the completion of whole animal tests (a small piece was cut from experimental substrates) using Ramé-Hart Instruments Advanced Goniometer 500 F1 with Drop Image Advanced software. Hexadecane contact angles were also measured relative to the smooth PDMSe substrates and used to replicate the gecko foot material in thermodynamic modeling (see discussion).

### Whole Animal Adhesion

Seven adult Tokay geckos (*Gekko gecko*) weighing on average of 98.48 ± 4.44g were used. Total toe pad area was 6.36 ± 0.41 cm^2^, averaged across all seven individuals. Toe pad area was measured using a flatbed scanner and ImageJ software (National Institutes of Health, Bethesda, MD, USA). The geckos were housed individually in glass terrariums, with 12 hours of UV light per day followed by 12 hours of dark. They were fed a diet of cockroaches three times per week and misted with water two times per day. Before each experimental trial geckos were acclimated to experimental conditions for 30 minutes. An ambient temperature of 23.5 ± 0.2°C, a humidity of 34.4 ± 0.7%, and a water temperature (if applicable) of 21.5 ± 0.2°C were maintained during the acclimation period and throughout trials. The University of Akron IACUC protocol 07-4G approved the procedures used on these live animals. The procedures were consistent with the guidelines provided by the Society for the Study of Amphibians and Reptiles (SSAR 2004).

A force sensing apparatus, described by Niewiarowski et al. [10], was used to measure the shear adhesive force of geckos standing horizontally in both air and in water on each substrate. Treatment condition (water or air), substrate (smooth, parallel, or perpendicular), and gecko were chosen at random. Two pelvic harnesses were used to attach the gecko to a force sensor. Once attached, the gecko was placed on the substrate and allowed to take one step with each foot, ensuring that the natural adhesive system of the gecko was being utilized. Geckos were then pulled backwards along the substrate on a motorized track. This allowed us to measure the maximum shear adhesive force produced by the gecko while clinging to the substrate. The maximum force was defined as the force reading at the point in time when all four of the gecko’s feet first slip along the substrate. The substrates were attached to the apparatus as described by Stark et al. [11], where a plastic container allowed us to test shear adhesion in water. The gecko’s feet were submerged in ~1cm of water while standing on the substrate. Geckos were not tested more than three times on a given day. When testing adhesion in water geckos were only tested once because if the feet become wet adhesive force is significantly reduced [9, 11]. Whether the gecko was tested three times or once, all geckos were given at least one day off between testing trials.

Three substrates were used in the experiment. The "parallel" substrate had the pattern described above oriented in a way that shear sliding occurred along the length of the substrate channels (Fig 2a). A second substrate was produced with a rotated pattern (90°), such that the pattern was perpendicular to the shearing direction of the gecko toe (Fig 2b). The last substrate was void of a pattern and acted as a control (termed "smooth"). Each gecko was tested three times in both air and in water on each of the three substrates. Only the highest maximum shear adhesive force of three tests was used in analysis.

### Statistical Analysis

We used a repeated measures MANOVA to test the overall effects of substrate (parallel, perpendicular or smooth) and treatment (air or water) on shear adhesion. To explore detailed effects subsequent to the MANOVA, we used a matched pairs analysis to separately compare shear adhesion in air and in water on each substrate. Finally, we used an ANOVA to test whether there were significant differences in shear adhesion across the three substrates in air and in water, using a Tukey HSD test to control for multiple comparisons. Each individual was tested in all combinations of treatments, which removes the need to account for differences across subjects. Shear adhesion was log transformed to comply with the model assumptions. Means are reported as mean ± 1 s.e.m.

## Results

**Fig 3:**
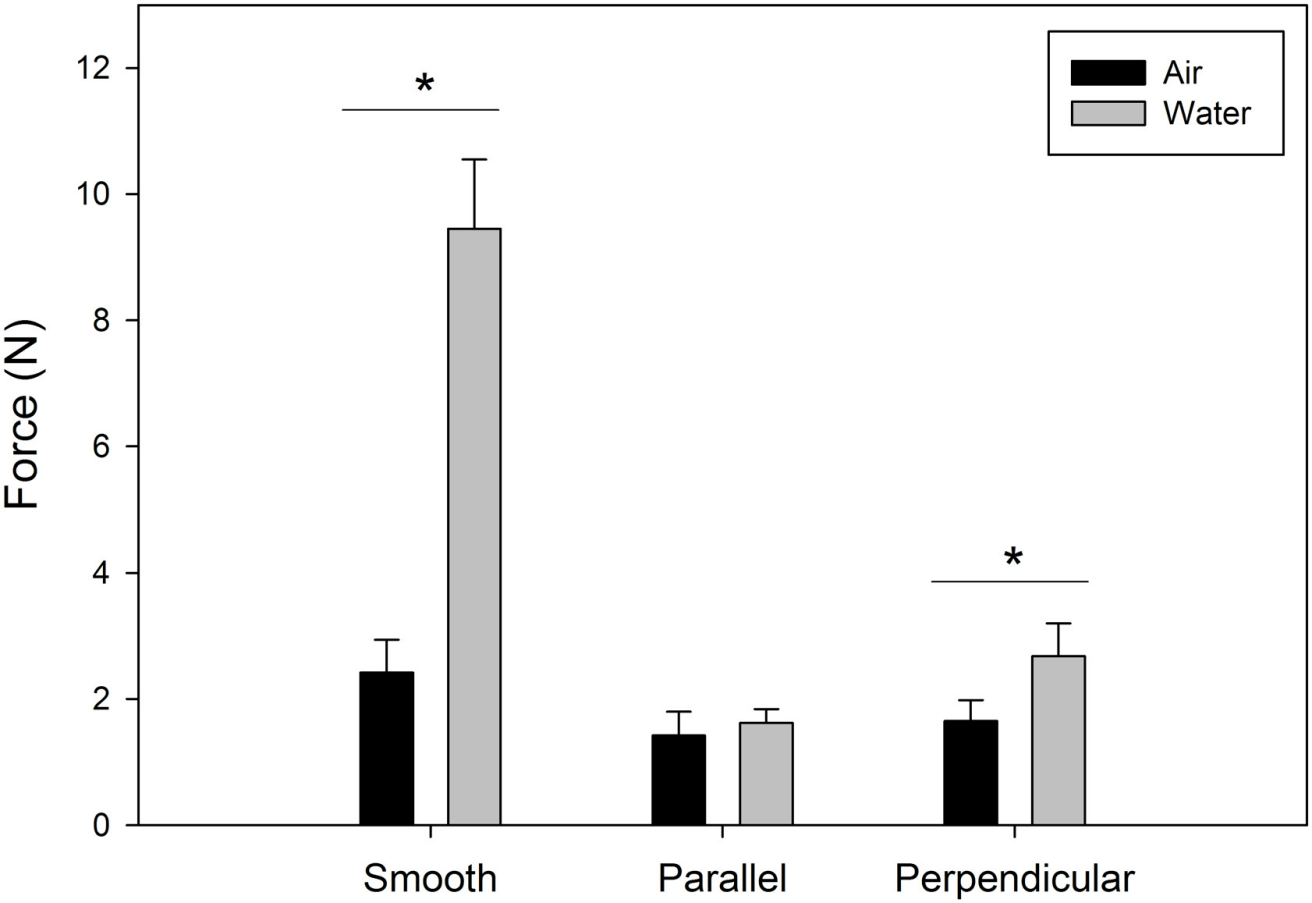
Shear adhesion of geckos on smooth and patterned substrates in air and water. Geckos were tested in air and in water on three substrates: smooth PDMSe (smooth), microstructured PDMSe oriented parallel to shear (parallel) and microstructured PDMSe oriented perpendicular to shear (perpendicular). Error bars are mean ± 1 s.e.m. The asterisk denotes a significant difference between the treatment groups (air and water). See Table 1 for details of statistical comparisons.

Shear adhesion was significantly affected by substrate (parallel, perpendicular and smooth; F_2,11_ = 230.902, p < 0.0001), treatment (air and water; F_1,12_ = 11.60, p = 0.0052) and their interaction (F_2,11_ = 60.407, p < 0.0001) (Table 1). The significant interaction arises because shear adhesion on the smooth and perpendicular substrate was significantly higher in water than in air (smooth, 9.45 ± 1.10N in water, 2.42 ± 0.52N in air, t = 7.58, df = 6, p = 0.0003; perpendicular, 2.68 ± 0.52N in water, 1.65 ± 0.33N in air, t = 2.67, df = 6, p = 0.0372), but there was no difference between water and air on the parallel substrate (1.62 ± 0.22N in water, 1.42 ± 0.38N in air, t = 0.88, df = 6, p = 0.4108) (Fig 3). Finally, there was no difference in shear adhesion across the three substrates when tested in air (F = 2.460, d.f. = 2, p = 0.1137), but when tested in water, the smooth substrate was significantly higher than the two patterned substrates (F = 36.510, d.f. = 2, p < 0.0001).

**Table 1:** Multivariate analysis of variance for the effect of substrate and treatment on gecko adhesion.

Effect	Wilks' Lambda	Exact F	Numerator d.f.	Denominator d.f	P value
Treatment	0.966	11.595	1	12	0.0052
Substrate	41.982	230.902	2	11	< 0.0001
Substrate X Treatment	10.983	60.407	2	11	< 0.0001

There is a significant difference in shear adhesion across substrate (parallel, perpendicular or smooth), treatment (air or water) and the interaction of substrate and treatment. The significant interaction arises because shear adhesion is sensitive to substrate in water but not in air (see results).

## Discussion

The adhesive system of geckos has been extensively studied and has inspired hundreds of synthetic mimics which can adhere in a variety of conditions [37]. Perhaps less well studied however, are the conditions that challenge the gecko adhesive system in their native environment. As with all biological organisms, geckos must counter multiple challenges at one time, like high temperature and humidity, surface water, substrate roughness and contamination. In order to investigate the interaction of multiple parameters on adhesion, we tested geckos on substrates with defined surface area and pattern, and in either air or water. We hypothesized that when tested on a smooth control substrate, adhesion would be equivalent in water and air due to the hydrophobic nature of the substrate [5]. When reducing available surface area however, we hypothesized that geckos would have reduced adhesion in air and water but that the pattern would have no impact on adhesion. Our results did not support our hypotheses. Instead, water enhanced adhesion on the smooth control substrate and the perpendicular substrate but not the parallel substrate.

When tested in air geckos adhered to all three substrates equally well, however, this was much lower than previous values on intermediately wetting and hydrophobic substrates (usually ~20N for whole animal studies) [5]. These results were surprising considering the contact angle of the smooth PDMSe substrate was hydrophobic, like that of glass coated with a hydrophobic octadecyltrichlorosilane self-assembled monolayer (OTS-SAM) used previously (contact angle for PDMSe = 115 ± 2° and OTS-SAM = 94 ± 0.5°) [5]. We do not expect any significant differences in substrate roughness between these two smooth substrates, which leads to two questions. First, how does the surface energy of PDMSe affect adhesion and second, how does the compliance of PDMSe affect adhesion? We will address the first question later in the discussion. The second question is intriguing. Gecko toes are soft, compliant structures. Thus, as this soft toe makes contact with a soft substrate, adhesion could be significantly affected. While the effect of substrate softness on gecko adhesion has never been tested directly, soft substrates exist in the native environment of geckos, therefore this question is potentially quite important when considering environmental challenges and possible constraints on adhesion. When investigating the effect of reduced surface area and pattern on adhesion in air, there was not a corresponding reduction in adhesion as hypothesized. Therefore, it is likely that the setae are able to conform to, or counter the dimensions of the substrate structure. To conclude, in air, adhesion is not disrupted by the Sharklet^®^ channels or orientation; and overall adhesion across all three substrates is reduced by a factor of 10 when qualitatively compared to a smooth, hard, hydrophobic substrate (OTS-SAM) [5].

Unlike adhesion in air, adhesion in water produced significant differences in shear adhesion across the three substrates. First, shear adhesion on the smooth substrate was significantly higher in water than in air. These values were still low compared to OTS-SAM in water (about half) [5]. We also found that the reduced surface area substrates produced significantly lower shear adhesion than the smooth in water, partially supporting our second hypothesis. These values were not different from one another, although it is interesting that when compared within substrate, adhesion on the perpendicular substrate was higher in water than air. Our results in water once again highlight the interesting behavior of the PDMSe substrate. On smooth PDMSe we expected water to be squeezed out of the contact interface by the hydrophobic material. Thus, adhesion in air should equal adhesion in water, and should not be significantly higher. Similar results (improved adhesion in water) were reported on polytetrafluoroethylene (PTFE), which is a rough (r.m.s. = ~230nm), hydrophobic substrate [5, 38]. Reduced adhesion did occur on the structured substrates, suggesting that water limits adhesion on substrates that contain pockets or channels at the size-scale tested here. We hypothesize this is due to filling of the channels by water, reducing available contact area and disrupting van der Waals forces. Surprisingly, pattern had a significant effect on within substrate adhesion, where the perpendicular substrate produced higher shear adhesives forces in water than air. This suggests that the pattern and direction of shear can play a role in gecko adhesion but only in water. As a gecko toe shears across the perpendicular substrate it moves in and out of the channels at a higher rate than when sliding along the parallel substrate (every 2μm rather than every 4–16μm depending on the channel; see Figs 1 and 2). It is possible that this behavior helps to expel water out of the contact interface more efficiently, or results in multiple stick-slip or contact line pinning events during sliding. Long et al. [39] observed similar pinning of the contact line during the measurements of advancing and receding contact lines.

While it is clear more work is necessary to understand how setae penetrate microscopic channels during dynamic shearing, it is possible to make some predictions. First, the channels in the structured substrates are only 2μm deep and 1.4 μm wide (Fig 1). Tokay gecko (*Gekko gecko*) setae are approximately 110μm long and branch several times before terminating into the nanometer-sized spatular structures [19]. Ruibal and Ernst [19] defined the final portion of branching in *G*. *gecko* as the quarternary branches. Quarternary branching begins approximately at 84μm from the base of the setae. Here the setae appear to branch one final time, bifurcating to terminate into the spatular pads. Thus the final ~26μm of the setae are thin fibrils with flattened pads which are about 200nm wide. Given this morphology it is likely the distal end of the setae have the compliance and size to penetrate into the shallow channels and make adhesive contact. This is supported by the results in air, where pattern had no affect on adhesion when compared to the smooth control. Second, using a 50μm^2^ grid we measured five random cells to estimate the average surface area available for gecko adhesion on the patterned substrates. We found that available surface area was reduced by about 34% with the addition of the pattern (33.13 ± 2.59 μm^2^ for the patterned substrate vs. 50μm^2^ for a smooth surface). Total toe pad area was 6.36 ± 0.41cm^2^, averaged across all seven individuals, thus a reduction of 34% leaves the gecko with approximately 4.20cm^2^ of toe pad area, more than enough to achieve maximal performance values we expect from whole animal adhesion [5, 10, 11, 40], even if we assume in this scenario that the setae do not penetrate the shallow channels. What is interesting however, is that in water the 34% reduction of surface area by shallow channels significantly impacts overall adhesion. When sliding in water the results can be complicated by structure (channels), location of water in the channels, surface energy and possibly compliance.

**Fig 4:**
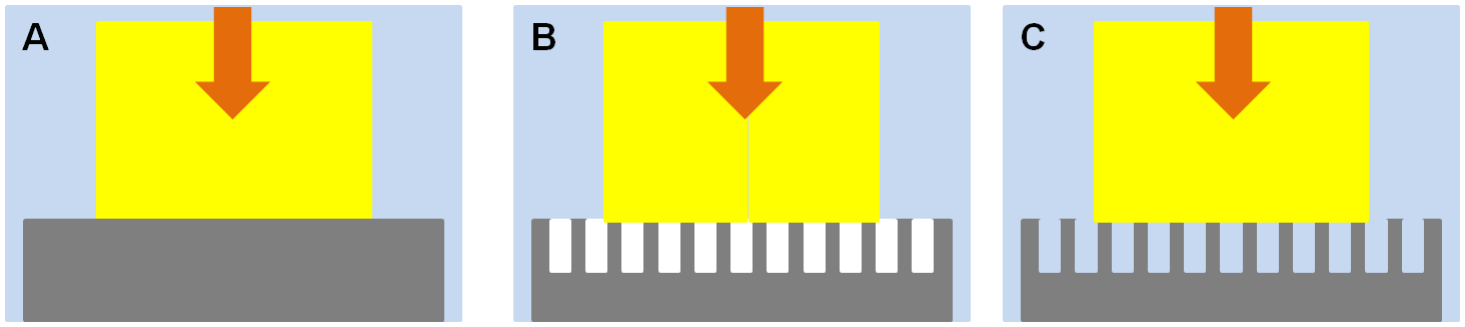
Schematic model of the contact made by a gecko toe on three substrates in water. Smooth PDMSe (A), structured PDMSe without water penetrating the surface asperities (i.e. air pockets) (B) and structured PDMSe penetrated by water (C) are displayed visually. Models are shown from the side. The generalized gecko foot is shown in yellow and all schematics are shown when making contact in water (blue). The arrow represents the direction of contact where the gecko foot is pressed into contact with the PDMSe (grey).

Deciphering the shear adhesion results in water will require knowledge of whether the contact interface between two contacting surfaces is dry or partially wet (as shown in Fig 4). The contact between two hydrophobic surfaces underwater was measured by Defante et al. [41] and showed that PDMS-PDMS contact underwater was dry and the wet normal adhesion was almost a factor of 2 greater than the dry contact. Because we know that lipids at least partially cover the contact interface in the natural system [5, 42, 43], which results in a hydrophobic or oil-like surface, we expect underwater adhesion to be higher than dry adhesion similar to the PDMS-PDMS contact. This is consistent with shear adhesion measurements of geckos in contact with the smooth PDMSe sheet. However, the dry contact model for a structured surface, with or without the channels filled with water (Fig 4B and 4C), predicts a factor of ~ 2.2 to 2.5 times higher underwater adhesion compared to the dry adhesion, though these models are based on a small sub-section of patterning where channels run the full length of the section and the model assumes normal rather than shear adhesion (for model calculations see [5, 44]). This indicates that for the structured surfaces, the contact interface is likely partially wet (Fig 3C) and water reduces underwater adhesion. It is also interesting to point out that these patterned surfaces are anti-fouling, and our data indicates that they are also difficult for geckos to grip.

To our knowledge this is the first experiment specifically designed to test whole-animal adhesion on substrates with controlled available surface area and pattern in the presence of air or water. Furthermore, the fabrication material used to mold the substrates is soft, adding an additional, unexpected parameter to our experiment. While our goal here was to test performance in a more complex and realistic series of conditions, there are several limitations related to the application of these results to the natural system. Principally, natural substrates are likely more variable than those used here. Surface roughness often varies at multiple scales (nanometer to centimeter), as does distribution of water and even surface chemistry (related to surface energy). Though patterns may exist in natural substrates, consider the vertical hills and channels of bark or the venous structure of leaves, they are certainly not as controlled as the substrates tested here. Furthermore, substrate modulus is highly variable and may consist of both hard and soft material, even over the course of one step. Our results do however highlight the importance of considering multiple parameters when investigating the gecko adhesive system. For instance, adhesion to the reduced surface area substrate was unaffected in air. In water however, both surface energy [5] and structure play a significant role in adhesion. Thus a gecko clinging to a rough, wet substrate should be more limited than one clinging to a wet smooth substrate, but not a dry substrate. Substrate parameters are not the only variables however. Geckos may locally change which substrates they use and when (i.e. after a rainstorm), or how they move across them. For instance, there is increasing evidence that factors like surface water are more limiting to static adhesion than the dynamic adhesion utilized during running [45]. There are also now several studies that relate specialized toe pad morphology to the substrates where geckos are primarily found, suggesting some drive toward adaptation to non-uniform, fundamentally challenging substrates [46–49]. In conclusion, our work highlights the need to investigate the effect of multiple parameters on gecko adhesion to improve our understanding of the natural system and potential constraints or drivers of specialized adaptations. Additionally, this work allows us to explore how these challenges are met by the natural system in order to improve our current synthetics, which may be tailored to adhere to wet, rough and soft substrates in the future.

## Supporting Information

S1 File Raw data. (XLSX)

## References

[1] Autumn K. How gecko toes stick—The powerful, fantastic adhesive used by geckos is made of nanoscale hairs that engage tiny forces, inspiring envy among human imitators. Am Sci. 2006;94(2):124–32. 10.1511/2006.58.124 ISI:000235380700028.

[2] Autumn K, Sitti M, Liang YCA, Peattie AM, Hansen WR, Sponberg S, et al Evidence for van der Waals adhesion in gecko setae. P Natl Acad Sci USA. 2002;99(19):12252–6. 10.1073/pnas.192252799 ISI:000178187000048. PMC129431 12198184

[3] Hansen WR, Autumn K. Evidence for self-cleaning in gecko setae. P Natl Acad Sci USA. 2005;102(2):385–9. 10.1073/pnas.0408304102 ISI:000226315700024. PMC544316 15630086

[4] Hu SH, Lopez S, Niewiarowski PH, Xia ZH. Dynamic self-cleaning in gecko setae via digital hyperextension. J R Soc Interface. 2012;9(76):2781–90. 10.1098/rsif.2012.0108 ISI:000309269100003. 22696482 PMC3479896

[5] Stark AY, Badge I, Wucinich NA, Sullivan TW, Niewiarowski PH, Dhinojwala A. Surface wettability plays a significant role in gecko adhesion underwater. P Natl Acad Sci USA. 2013;110(16):6340–5. 10.1073/pnas.1219317110 ISI:000318041500032. PMC3631644 23576727

[6] Gillies AG, Henry A, Lin H, Ren A, Shiuan K, Fearing RS, et al Gecko toe and lamellar shear adhesion on macroscopic, engineered rough surfaces. The Journal of Experimental Biology. 2014;217(2):283–9. 24115057 10.1242/jeb.092015

[7] Vanhooydonck B, Andronescu A, Herrel A, Irschick DJ. Effects of substrate structure on speed and acceleration capacity in climbing geckos. Biol J Linn Soc. 2005;85(3):385–93. 10.1111/j.1095-8312.2005.00495.x

[8] Zani P. The comparative evolution of lizard claw and toe morphology and clinging performance. J Evolution Biol. 2000;13(2):316–25.

[9] Stark AY, Wucinich NA, Paoloni EL, Niewiarowski PH, Dhinojwala A. Self-Drying: A Gecko's Innate Ability to Remove Water from Wet Toe Pads. Plos One. 2014;9(7). doi: ARTN e101885 10.1371/journal.pone.0101885 ISI:000339614100021. PMC4108337 25054217

[10] Niewiarowski PH, Lopez S, Ge L, Hagan E, Dhinojwala A. Sticky Gecko Feet: The Role of Temperature and Humidity. Plos One. 2008;3(5). doi: Artn E2192 10.1371/Journal.Pone.0002192 ISI:000262172800056. PMC2364659 18478106

[11] Stark AY, Sullivan TW, Niewiarowski PH. The effect of surface water and wetting on gecko adhesion. J Exp Biol. 2012;215(17):3080–6. 10.1242/Jeb.070912 ISI:000307638700026. 22875772

[12] Losos J. Thermal sensitivity of sprinting and clinging performance in the Tokay gecko (Gekko gecko). Asiatic Herpetological Research. 1990;3:54–9.

[13] Bergmann PJ, Irschick DJ. Effects of temperature on maximum clinging ability in a diurnal gecko: evidence for a passive clinging mechanism? Journal of Experimental Zoology Part A: Comparative Experimental Biology. 2005;303(9):785–91. 10.1002/jez.a.210 16106405

[14] Stark AY, McClung B, Niewiarowski PH, Dhinojwala A. Reduction of Water Surface Tension Significantly Impacts Gecko Adhesion Underwater. Integr Comp Biol. 2014;54(6):1026–33. 10.1093/icb/icu066 24944119

[15] Russell AP. A contribution to the functional analysis of the foot of the Tokay, Gekko gecko (Reptilia: Gekkonidae). J Zool. 1975;176(4):437–76. 10.1111/j.1469-7998.1975.tb03215.x

[16] Russell AP. The morphological basis of weight-bearing in the scansors of the tokay gecko (Reptilia: Sauria). Canadian Journal of Zoology. 1986;64(4):948–55. 10.1139/z86-144

[17] Russell AP. Descriptive and functional anatomy of the digital vascular system of the tokay, Gekko gecko. J Morphol. 1981;169(3):293–323. 10.1002/jmor.1051690305 30114860

[18] Russell AP. Integrative Functional Morphology of the Gekkotan Adhesive System (Reptilia: Gekkota). Integr Comp Biol. 2002;42(6):1154–63. 10.1093/icb/42.6.1154 21680400

[19] Ruibal R, Ernst V. The structure of the digital setae of lizards. J Morphol. 1965;117(3):271–93. 5883924 10.1002/jmor.1051170302

[20] Maderson P. Keratinized epidermal derivatives as an aid to climbing in gekkonid lizards. Nature. 1964;203:780–781.

[21] Williams E, Peterson J. Convergent and alternative designs in the digital adhesive pads of scincid lizards. Science. 1982;215(4539):1509–11. 17788677 10.1126/science.215.4539.1509

[22] Rizzo N, Gardner K, Walls Dea, Keiper-Hrynko N, Ganzke T, Hallahan D. Characterization of the structure and composition of gecko adhesive setae. J R Soc Interface. 2006;3(8):441–51. 16849272 10.1098/rsif.2005.0097 PMC1578751

[23] Autumn K, Majidi C, Groff RE, Dittmore A, Fearing R. Effective elastic modulus of isolated gecko setal arrays. J Exp Biol. 2006;209(18):3558–68. 10.1242/Jeb.02469 ISI:000240154200009. 16943496

[24] Huber G, Gorb SN, Hosoda N, Spolenak R, Arzt E. Influence of surface roughness on gecko adhesion. Acta Biomater. 2007;3(4):607–10. 10.1016/j.actbio.2007.01.007 ISI:000247865100020. 17376751

[25] Pugno NM, Lepore E. Observation of optimal gecko's adhesion on nanorough surfaces. Biosystems. 2008;94(3):218–22. 10.1016/j.biosystems.2008.06.009 18718501

[26] Pugno NM, Lepore E. Living tokay geckos display adhesion times following Weibull Statistics. The Journal of Adhesion. 2008;84(11):947–60.

[27] Lepore E, Brianza S, Antoniolli F, Buono M, Carpinteri A, Pugno N. Preliminary in vivo experiments on adhesion of geckos. Journal of Nanomaterials. 2008;2008.

[28] Russell AP, Johnson MK. Between a rock and a soft place: microtopography of the locomotor substrate and the morphology of the setal fields of Namibian day geckos (Gekkota: Gekkonidae: Rhoptropus). Acta Zool-Stockholm. 2014;95(3):299–318.

[29] Endlein T, Barnes WJP, Samuel DS, Crawford NA, Biaw AB, Grafe U. Sticking under Wet Conditions: The Remarkable Attachment Abilities of the Torrent Frog, Staurois guttatus. Plos One. 2013;8(9). doi: ARTN e73810 10.1371/journal.pone.0073810 ISI:000325218700013. PMC3783468 24086297

[30] Green DM, Barber DL. The ventral adhesive disc of the clingfish Gobiesox maeandricus: integumental structure and adhesive mechanisms. Canadian Journal of Zoology. 1988;66(7):1610–9. 10.1139/z88-235

[31] Ditsche P, Wainwright DK, Summers AP. Attachment to challenging substrates—fouling, roughness and limits of adhesion in the northern clingfish (Gobiesox maeandricus). The Journal of Experimental Biology. 2014;217(14):2548–54. 10.1242/jeb.100149 25031458

[32] Wainwright DK, Kleinteich T, Kleinteich A, Gorb SN, Summers AP. Stick tight: suction adhesion on irregular surfaces in the northern clingfish 2013 2013-06-23 00:00:00. 10.1098/rsbl.2013.0234 PMC3645053 23637393

[33] Ditsche-Kuru P, Koop JHE, Gorb SN. Underwater attachment in current: the role of setose attachment structures on the gills of the mayfly larvae Epeorus assimilis (Ephemeroptera, Heptageniidae). The Journal of Experimental Biology. 2010;213(11):1950–9. 10.1242/jeb.037218 20472782

[34] Chung KK, Schumacher JF, Sampson EM, Burne RA, Antonelli PJ, Brennan AB. Impact of engineered surface microtopography on biofilm formation of Staphylococcus aureus. Biointerphases. 2007;2(2):89–94. 10.1116/1.2751405 20408641

[35] Schumacher JF, Long CJ, Callow ME, Finlay JA, Callow JA, Brennan AB. Engineered Nanoforce Gradients for Inhibition of Settlement (Attachment) of Swimming Algal Spores. Langmuir. 2008;24(9):4931–7. 10.1021/la703421v 18361532

[36] Magin CM, Cooper SP, Brennan AB. Non-toxic antifouling strategies. Materials Today. 2010;13(4):36–44. 10.1016/S1369-7021(10)70058-4

[37] Boesel LF, Greiner C, Arzt E, del Campo A. Gecko-Inspired Surfaces: A Path to Strong and Reversible Dry Adhesives. Adv Mater. 2010;22(19):2125–37. 10.1002/adma.200903200 20349430

[38] Stark AY, Dryden DM, Olderman J, Peterson KA, Niewiarowski PH, French RH, et al Adhesive interactions of geckos with wet and dry fluoropolymer substrates. J R Soc Interface. 2015;12(108). 10.1098/rsif.2015.0464 PMC4528608 26109635

[39] Long CJ, Schumacher JF, Brennan AB. Potential for Tunable Static and Dynamic Contact Angle Anisotropy on Gradient Microscale Patterned Topographies. Langmuir. 2009;25(22):12982–9. 10.1021/La901836w ISI:000271522800023. 19603771

[40] Irschick DJ, Austin CC, Petren K, Fisher RN, Losos JB, Ellers O. A comparative analysis of clinging ability among pad-bearing lizards. Biol J Linn Soc. 1996;59(1):21–35. 10.1111/j.1095-8312.1996.tb01451.x

[41] Defante AP, Burai TN, Becker ML, Dhinojwala A. Consequences of Water between Two Hydrophobic Surfaces on Adhesion and Wetting. Langmuir. 2015;31(8):2398–406. 10.1021/la504564w 25668056

[42] Hsu PY, Ge LH, Li XP, Stark AY, Wesdemiotis C, Niewiarowski PH, et al Direct evidence of phospholipids in gecko footprints and spatula-substrate contact interface detected using surface-sensitive spectroscopy. J R Soc Interface. 2012;9(69):657–64. 10.1098/rsif.2011.0370 ISI:000300726700006. 21865250 PMC3284128

[43] Jain D, Stark AY, Niewiarowski PH, Miyoshi T, Dhinojwala A. NMR spectroscopy reveals the presence and association of lipids and keratin in adhesive gecko setae. Sci Rep-Uk. 2015;5. doi: Artn 9594 10.1038/Srep09594 ISI:000353283000001. PMC5386106 25902194

[44] Badge I, Stark AY, Paoloni EL, Niewiarowski PH, Dhinojwala A. The Role of Surface Chemistry in Adhesion and Wetting of Gecko Toe Pads. Sci Rep-Uk. 2014;4. doi: Artn 6643 10.1038/Srep06643 ISI:000343294000001. PMC4200409 25323067

[45] Stark AY, Ohlemacher J, Knight A, Niewiarowski PH. Run don't walk: locomotor performance of geckos on wet substrates. The Journal of Experimental Biology. 2015 10.1242/jeb.120683 26034124

[46] Johnson MK, Russell AP. Configuration of the setal fields of Rhoptropus (Gekkota: Gekkonidae): functional, evolutionary, ecological and phylogenetic implications of observed pattern. J Anat. 2009;214(6):937–55. 10.1111/j.1469-7580.2009.01075.x 19538637 PMC2705302

[47] Collins CE, Russell AP, Higham TE. Subdigital adhesive pad morphology varies in relation to structural habitat use in the Namib Day Gecko. Funct Ecol. 2015;29(1):66–77. 10.1111/1365-2435.12312

[48] Russell A, Johnson M. Real-world challenges to, and capabilities of, the gekkotan adhesive system: contrasting the rough and the smooth. Canadian Journal of Zoology. 2007;85(12):1228–38.

[49] Higham TE, Russell AP. Divergence in locomotor performance, ecology, and morphology between two sympatric sister species of desert-dwelling gecko. Biol J Linn Soc. 2010;101(4):860–9. 10.1111/j.1095-8312.2010.01539.x

